# Enhancing Person-Centered Audiologic Rehabilitation: Exploring the Use of an Interview Tool Based on the International Classification of Functioning, Disability, and Health Core Sets for Hearing Loss

**DOI:** 10.3389/fresc.2022.945464

**Published:** 2022-07-13

**Authors:** Sarah Granberg, Åsa Skagerstrand

**Affiliations:** ^1^School of Health Sciences, Faculty of Medicine and Health, Örebro University, Örebro, Sweden; ^2^Audiological Research Center, Faculty of Medicine and Health, Örebro University, Örebro, Sweden; ^3^Department of Audiology, Faculty of Medicine and Health, Örebro University, Örebro, Sweden

**Keywords:** ICF (International Classification of Functioning, Disability and Health), hearing loss, audiologic rehabilitation (AR), person-centered, interview tool

## Abstract

Health care interventions that are intended to improve hearing should be based on the results of individual patient assessments. To improve these assessments, the feasibility of an International Classification of Functioning, Disability, and Health (ICF)-based interview tool was tested in a single clinical setting in Sweden. Audiologists participating in the study used the interview tool during a four-week testing period and provided written reflections after each session. The use of this tool was also evaluated in a focus group interview that took place after the completion of the project. The results of this study identified both process-related and structure-related factors that were highly relevant to the implementation of this interview tool. Overall, the findings revealed that the use of this interview tool promoted person-centered care in encounters focused on clinical audiological rehabilitation. Specifically, the ICF-based holistic approach permitted the audiologists to acquire more comprehensive patient narratives. The use of the ICF interview tool facilitated patient participation and permitted the audiologist to collect more substantial and meaningful information from each patient.

## Introduction

The World Health Organization (WHO) estimates that approximately 20% of the global population currently suffers from some degree of hearing loss. Hearing loss is likely to increase and is anticipated to have an impact on 25% of the population by 2050; thus, approximately 7% of the global population will require hearing health care (1).

While the etiology of this disorder varies, sensorineural hearing losses are dominant in the adult population (2, 3). Damages in the inner ear and/or the central auditory system results in sensorineural hearing loss. These losses result in a diminished ability to detect and comprehend sounds that are regarded as normally perceivable. Sensorineural hearing loss also has an impact on the quality of heard sounds, for example, speech that is perceived as unclear or distorted. While external hearing aids are the most commonly provided hearing intervention for these patients, this intervention alone is insufficient as it does not fully facilitate activities of daily living for adults with hearing loss (4). Because of the nature of this health condition and the increased demands in society for sufficient hearing and communication, individual consequences of hearing and communication deficits may include reduced quality of life (5), pain in the head, shoulders, and neck (6, 7), and fatigue (8), as well as reduced ability to work (9) and to participate in social activities (10) thus leading to social isolation (11). Hence, many adults experience hearing loss as very disabling.

Literature on adult audiologic rehabilitation (AR) published during the past 5 years has focused to a large extent on a person-centered perspective. This perspective acknowledges the complexity of hearing loss and advises the use of a bio-psycho-social approach to person-centered AR (12). This represents a significant shift from a more medically-focused AR and interventions focused primarily on the hearing impairment to a more holistic view that targets interventions based on functioning (i.e., bodily aspects, daily life activities, and the environment of a given individual).

The International Classification of Functioning, Disability, and Health (ICF) core sets for hearing loss were published in 2013 (13). Rigorous empirical and literature-based evidence (14–17) was used to develop two core sets (comprehensive and brief) that included 117 and 27 categories, respectively. Because the ICF core sets for hearing loss address functioning, they have contributed significantly to person-centered care and research literature. The brief ICF core sets for hearing loss demonstrate appropriate validity for use in international settings (18).

Hearing health care in Sweden is taxpayer-funded. AR is provided by licensed audiologists who are required to base their practice on state-of-the-art scientific knowledge (19). Typically, AR in Sweden include provision of hearing aids and assistive listening devices, and communication strategies training. To enhance the person-centered focus in AR clinical encounters, Skagerstrand has developed an interview tool (2021, unpublished) based on the ICF core sets for hearing loss that can be used by audiologists during the initial patient interview (see methods section). Given the premises for working as an audiologist in Sweden, this tool is designed to provide a scientific basis for the content of the initial patient interview and ensure that all relevant aspects of individual functioning are recognized by the clinician. The information collected during the patient interview will then be used to guide the audiologist toward adequate and appropriate AR interventions.

The objective of this study was to explore the feasibility of this ICF-based interview tool by exploring its use by a sample group of audiologists engaged in AR-clinical encounters.

## Methods

This work was designed as a feasibility study executed between February and April 2022. As recommended by Bowen et al. (20), the study employed a post-only design including structured reflections and a focus-group session. This study followed the evaluation criteria by Patton (21) to ensure credibility of the research including, e.g., accurate methods for data collection and analysis, and sufficient descriptions of the results.

### Participants and Setting

The study was carried out at the Audiological Clinic in Region Örebro County, one of 21 counties in Sweden. This is an umbrella clinic that provides broad audiological services, including aural habilitation and AR for children and adults, respectively. The clinic is divided into three subunits, located in three different cities within the region, that serve a total of 307,000 inhabitants. Audiologists represent the largest professional category of service providers at this clinic; these individuals usually practice AR independently. AR is typically provided in a series of three to five appointments with an audiologist. An individual interview is performed during the first appointment. The audiologist then collaborates with the patient to design a rehabilitation plan based on the outcomes of the interview.

In the current study, four licensed audiologists employed at the aforementioned umbrella clinic performed individual interviews with AR patients using the ICF-based interview tool. The four audiologists have an average experience of 9.5 years (range, 1–16 years).

### ICF-Based Interview Tool

The interview tool includes eight main areas of initial focus based on ICF core sets for hearing loss that include physical health, psychological health, daily activities, communication, hearing issues, living habits, living environment, and motivation to rehabilitation. The relationship between these eight areas and the ICF categories are as shown in Table 1. Of note, the tool featured specific ICF categories but did not include ICF category codes. A topic focused on “living habits” was implemented in the interview tool because of legal aspects and previous evidence indicating its relevance (22–25).

**Table 1:** Areas of the ICF-based interview tool and corresponding ICF categories.

**Areas**	**Corresponding ICF categories**
Physical health	b210 Seeing functions b280 Sensation of pain b710 Mobility of joint functions b780 Sensations related to muscles and movement functions e355 Health professionals * e580 Health services, systems and policies*
Psychological health (including mental functions)	b126 Temperament and personality functions b140 Attention functions b144 Memory functions b152 Emotional functions b164 Higher-level cognitive functions d240 Handling stress and other psychological demands
Daily activities	d740 Formal relationships d750 Informal relationships d830 Higher education d850 Remunerative employment d910 Community life d920 Recreation and leisure
Hearing issues	b230 Hearing functions b240 Sensations associated with hearing and vestibular functions
Communication (including facilitating/hindering environmental factors)	d115 Listening d310 Communication with -receiving- spoken messages d3503 Conversing with one person d3504 Conversing with many people d3600 Using telecommunication devices d3602 Using communication techniques e1250 General products and technology for communication e1251 Assistive products and technology for communication e250 Sound e360 Other professionals (interpreters)
Living habits	b1300 Energy level b134 Sleep functions d5701 Managing diet and fitness d5702 Maintaining one's health
Living environment	d760 Family relationships e310 Immediate family e315 Extended family e325 Acquaintances, peers, colleagues, neighbors and community e410 Individual attitudes of immediate family members e425 Individual attitudes of acquaintances, peers, colleagues, neighbors and community e460 Societal attitudes
Motivation	Pf Motivation towards AR

^*^The impact of health professionals and health services, systems and policies were specifically evaluated in relation to the patients overall physical health.

### Materials for Data Collection

Data were collected from both reflection protocols and a group interview.

### Reflection Protocol

The reflection protocol was based on Gibbs' reflective cycle model (26). This systematic reflection model presents a framework for structured reflections on repeated experiences and permits the user to learn and evolve by identifying aspects that were or were not effective in specific situations and experiences. In addition to the reflection protocol, the audiologists were asked to consider whether their current professional training provided them with sufficient insight into how to use interview tool or if they recognized areas beneficial for additional training.

### Group Interview

A group interview held with the participating audiologists focused on four aspects, including (a) the overall experience with the ICF-based interview tool, (b) the content of the tool, (c) the reflections and the reflection protocol, and (d) rehabilitation plans and interventions.

The group interview was moderated by the first author (SG); the second author (ÅS) took notes and assisted with the interview process. The session lasted 1 h and 34 min and was voice recorded by two separate devices to enhance the security and accuracy of the data collection.

### Procedure

The study was carried out in three phases, a pre-implementation phase, an implementation phase, and a post-implementation phase.

The pre-implementation phase provided education to the audiologists who were participating in the project. The education provided included lectures focused on the principles of ICF, the ICF core sets, and the specific content of the ICF-based interview tool. The education also focused on interview skills, including a general discussion of how to perform person-centered interviews as well as the content and use of the reflection protocol.

During the implementation phase, the audiologists carried out individual interviews of patients in AR using the ICF-based interview tool. Each patient session included an interview, rehabilitation planning, and, once the patient had left, reflections based on the reflection protocol and written notes placed in the patient's medical chart. After each patient interview, the audiologist posted written reflections in a secure data map maintained by the clinic that could be accessed only by the participating audiologists and the authors of the study. The four audiologists completed 25 individual interviews during the period of investigation.

The group interview was held post-implementation after all the patient interviews were completed and all reflections posted. The four audiologists and the two authors of this study participated in the group interview.

### Analysis

All data were evaluated by qualitative thematic analysis (27). The analysis included six specific steps that involved (1) reviewing the entire dataset, (2) generating initial codes, (3) searching for themes, (4) reviewing the chosen themes, (5) defining and naming the themes, and (6) generating the report.

The first author (SG) performed steps 1 and 2 as part of the analysis of the recorded interview. The second author (ÅS) performed steps 1 and 2 as part of the analysis of the reflection protocols. The data from the reflection protocols and the interview were then merged and steps 3–6 were conducted in collaboration between the two authors.

In addition to the thematic analysis, the two authors provided a joint general assessment of the outcomes of the reflection protocols. This assessment focused specifically on the learning curve, i.e., whether progression regarding the use of the interview instrument and protocol for patients in AR could be detected.

### Ethics

This study followed “Good research practice” outlined by the Swedish Research Council. However, as this project did not collect sensitive personal information, no formal approval from an ethics committee was required (28).

## Results

**Figure 1:**
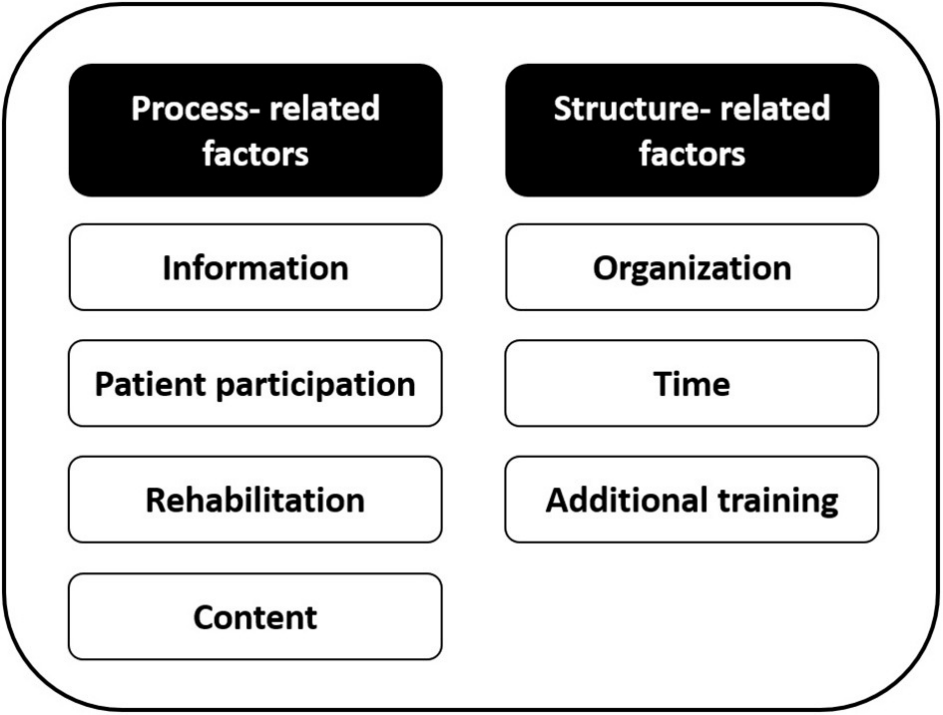
Overview of overarching themes with sub-themes.

The two overarching themes with specific relevance to feasibility emerged from the data analysis, including process-related and structure-related factors; both included several sub-themes (Figure 1).

### Process-Related Factors

Process-related factors include sub-themes that focus on the interaction between patients and the audiologists.

### Information

The general opinion among the audiologists was that they were able to collect deep and important information from each patient that might not have surfaced in the absence of this interview tool. The audiologists also identified two major obstacles. First, they reported unfamiliarity what “to do” with all the information. Examples of these issues included patient reports of smoking, sleep, and diet; the audiologists reported that it was difficult to discuss these health concerns while focusing on hearing loss. However, during the group interview, the four audiologists collectively determined ways that they might address these matters with their patients and agreed upon several solutions. They concluded that discussions of these aspects with the patient frequently led to their improved understanding of the complexities of living with hearing loss. The audiologists also noted the lack of routines for referral of the patients to other health care professionals and/or how to manage relevant aspects of patient care that did not relate specifically to the practice of the audiology.

As a second obstacle, the audiologists reported difficulties in managing some of the patients' emotional responses to discussions of living with hearing loss. They reported that they did not know how to “stop” patients at an appropriate place in these discussions. One of them said:

…*one of my patients opened up a lot… I had difficulties to meet this information…a heavy session…afterwards I thought, I cannot work this way, I feel totally drained…*

### Patient Participation

The audiologists note that they realized rather quickly that it was helpful to educate the patients by sharing the pre-printed figure that outlined the use of the interview tool. The pre-printed circle-type figure included content in the interview tool in a form that would facilitate its use by the audiologists. According to the audiologists, the patients responded positively to this pedagogic activity, as it permitted them to follow the progression of the interview and return to previous themes to provide additional information. The audiologists highlighted that they were able to provide a better understanding of the holistic view of hearing loss to patients engaged in this deeper type of interview.

…*a patient might wonder, “what has this to do with my hearing” and then I explained, and we discussed. I usually do not do this, I mean explain so deeply, but now when I do, they clearly open up and the dialog between us become good…*

The audiologists stated clearly that the interview tool enhanced patient participation because of its holistic content. They noted that several patients reported that the audiologists listened to their concerns, presented a more in-depth understanding of their current life situation, and met their narratives adequately.

### Rehabilitation

The audiologists reported that the additional information obtained from the patients did not result in any extended AR beyond hearing aids and interventions related to communication and that the rehabilitation plans formulated in collaboration with the patients were largely the same as those generated before initiating the project. One audiologist explained that the generated plan was based on the professional focus, i.e., primarily hearing technology and communication strategies. This interesting finding revealed some uncertainty regarding the full nature of a rehabilitation plan and the information that it should contain. It was clear that the audiologists did not fully understand that their advice to patients, recommendations, and patient support were extremely important aspects of the rehabilitation program that served to empower patients and facilitate their daily lives. When discussing this point during the interview, all four audiologists agreed that they are engaged in a great deal of rehabilitation work that is ultimately not disclosed in rehabilitation plans and/or presented in medical records. They agreed that if they had documented everything that they actually had done during the patient visit, there would be a better match between the information collected during the interviews and the documented rehabilitation plan. They also agreed that because the document with the rehabilitation plan is available to patients during the entire rehabilitation period, the inclusion of topics discussed in the interview (i.e., sleep habits, rest, and recovery) would provide the patients with important cues and reminders. They also noted that it is important to provide more complete documentation of patient care provided in the clinic so this information would be available for professional evaluation by management and oversight boards.

### Content

The audiologists reported that it was easy to discuss the content of the interview tool with the patient as the information provided closely resembled the “common view” of hearing loss as it focused on hearing, hearing comorbidities, communication, and the living situation. They also reported that the patients had no trouble when asked to discuss these matters. By contrast, all four audiologists struggled with aspects of the interview that were related to hearing loss but that did not fit the “common view,” especially those that highlighted physical health, psychological health, and factors related to lifestyle. As noted above, they felt unable to respond appropriately to the patient narratives and to provide adequate advice regarding the psychological aspects of hearing loss. They also struggled to identify the appropriate “level” of information. This was of particular concern for questions regarding physical health as many patients were eager to share detailed information. Nevertheless, they clearly realized the importance of this information. One audiologist said:

…*I had a patient who recently had learned that* XXX *had* [another diagnosis] *and I asked whether this was the right time to engage in AR given that it takes lots of energy and dedication to adapt to hearing aids. I offered* XXX *to think about it and we booked a new appointment, but I was very clear that* XXX *must consider what health condition to deal with first and to call me and reschedule should* XXX *not have the strength to deal with the hearing right now…*

### Structure-Related Factors

Structure-related factors include the context of, and aspects related to, the conditions needed to carry out AR.

### Organization

One concern noted by the audiologists was that AR was evaluated clinically by the number of patients and the number of hearing aid prescriptions provided over time and not by measurements that assess the quality of their work. They argued that these issues complicated their efforts to provide person-centered care. Likewise, while they reported previous positive experiences with patient follow-ups (typically within 6–12 months after completion of the AR intervention), these types of appointments have been discouraged by the present organization because of the long waiting lists for patients in need of primary hearing health care. The audiologists reported that follow-ups are important for most patients as it facilitates reinforcement of critical information, communication strategies, and the handling of technical aids. They also agreed that, in the absence of patient follow-up visits, it becomes difficult to evaluate the outcome and the quality of the primary interventions and to evaluate the overall impact of AR.

…*I think it is useful* [follow-ups] *because one can really grasp what* [in AR] *that have been absorbed by the patients and what we have to work with further…*

### Time

Although the audiologists considered the interview tool beneficial for patient interviews, there were some concerns about time requirements. The ICF-based interviews were more in-depth and thus they were more time-consuming, both with respect to direct patient interaction and documentation of findings in the medical record. The audiologists report that they are time-limited in their regular daily work due to the number of aspects and tasks that need to be fulfilled for each patient and at each appointment. This was identified as a significant hindrance to person-centered AR within the current organizational structure. As stated by one of the audiologists:

…*people have more problems today, we have more technology to give information about, it is not just the interview, but we inform about hearing aids, compatibilities between hearing aids and cell phones etc. It takes a lot of time to see if they can handle it* [the technology], *the patients expect us to do more* [concerning technology]. *That takes time…*

### Additional Training

Questions in the reflection tool revealed the need for additional training. The audiologists reported the need for both informal collegial interactions as well as more formal training sessions. The audiologists noted that they did not have the opportunity to engage in structured collegial discussions that might provide insights on how to manage the daily assignments most effectively. They also raised the need for improved knowledge regarding ways to respond to cognitive and psychological issues and how to address the specific needs of patients who remain in the workforce.

### Learning Curve

The interview tool appears to be beneficial as it enhances the nature and quality of the initial patient interview. The findings suggest that improved structure and flow of the interviews were achieved; the analysis of the structured reflections also demonstrated improvement over time. When first introduced, the new interview format was stressful for the audiologists. However, over time, the audiologists became more confident in their skills and were able to focus on the information provided by the patient rather than the interview tool itself. Furthermore, the reflections revealed a change in the depth of information gathered from each patient. As the audiologists became more experienced with the interview tool, they noted that it became easier to address patient concerns in-depth and to cover more areas that may be relevant to the AR process. One interesting observation was the change in the way audiologists used the interview tool. At the beginning of the trial, it was perceived as a tool for the audiologists only; by the end of the project, many of the audiologists shared this tool in collaboration with the patients.

## Discussion

The current project evaluated the feasibility of an ICF-based interview tool to enhance person-centered AR. The data analysis supports the use of this tool in clinical encounters with patients in need of AR.

### Use of ICF to Ensure a Holistic View for Patients in AR

The interview tool developed for this project featured content from the ICF Core sets for hearing loss but not the numerical codes. The use of these codes requires a medical record system that facilitates the use of keywords related to these codes as well as in-depth knowledge that is not currently provided to most trained audiologists. However, the interview tool based on the principles of ICF ensure a holistic view of patient function. This finding is in line with the results from a previous study that demonstrated an overlap between categories in the brief ICF core sets for hearing loss and information in medical records (29). Hence, the interview tool in the current study might also be useful for audiologists who do not have access to medical records but wishes to work holistically in AR. Multiple functional aspects may be associated with hearing loss in adults (30) and can have a broad impact on patients living with hearing loss. Furthermore, many of these aspects need to be included in the AR program to empower the patients and facilitate their activities of daily living. ICF has been acknowledged widely in the literature as a holistic framework that facilitates person-centered AR. Numerous studies have concluded that a holistically based view such as that provided by ICF is necessary to address all relevant aspects of adult hearing loss in AR (4, 12, 29, 31). Saunders et al. [(32), p. S86] concluded that a “redefinition of therapeutic goal setting and hearing outcomes to include aspects of well-being so that audiologists can capture and patients realize that good hearing outcomes can have a direct positive impact on a person's quality of life that extends beyond their improved ability to hear” will be necessary for clinical practice to facilitate well-being and healthy living. The audiologists participating in this study worked in a person-centered way, and thus, could incorporate the ICF-based interview tool in their daily practices. All four audiologists highlighted patient participation as a core outcome of this project. Consequently, we can conclude that the interview tool promoted a holistic view aligned with both person-centered AR and a major aim of ICF, i.e., to be used as a conceptual basis for disability and health (33).

The interview tool was based on the ICF core set for hearing loss with the addition of questions focused on lifestyle factors. This addition was made in accordance with Swedish regulations that require professional health care providers to acknowledge lifestyle factors and their role in improving health (23). The audiologists participating in this project reported some difficulties with these questions, due to inexperience with the routines of the referral process. This finding suggests that they may need to identify possibilities to explore and incorporate new research findings as part of their daily work.

### Organizational Aspects That Facilitate Implementation of the ICF Based Interview-Tool

Santana et al. (34) described several fundamental aspects of person-centered care that relate specifically to structures and organizations. Organizations and management play critical roles in establishing policies and practices and fostering a person-centered work climate among the health care providers and staff. The audiologists participating in this project were not aware of any policies or practices that were directly related to person-centered care nor were these qualities considered in their professional evaluations. This arrangement is clearly in conflict with person-centered ideas and practices. Thus, when implementing the ICF-based interview tool, one must also focus on the concerns of management and the overall organization. The management has the power and the responsibility to create structures that facilitate person-centered AR. Santana et al. also remind us that changes in the health care curriculum do not automatically result in cultural changes within a given clinic. These findings suggest that change cannot be implemented by single health care professionals; all staff members must be involved to achieve success.

### Structured Reflections

The progressive facility using the interview tool was clearly related to increased audiologist familiarity and confidence. These findings suggest that, when implementing the use of an interview tool, it is useful to include a training period involving structured reflections. Structured reflections provide the user with the tools needed to learn and evolve (26). This type of activity provides essential contributions to those engaged in professional development and learning. Similarly, a structured implementation, as shown in this project, may be necessary to support the successful use of new interventions and work habits (35, 36). Our results clearly demonstrate that audiologists have both the need and desire to improve their knowledge base. This desire is probably not unique to this group or this clinical setting and is more likely to be a sign of increasingly complex needs within the area of hearing health care. The field of audiology is undergoing constant evolution with needs that may be met by ongoing updates designed specifically for audiology professionals.

## Conclusion

The current study explored the feasibility of an ICF-based interview tool for use in patients in need of AR. The two overarching themes identified focused on process-related and structure-related factors. Our analysis revealed that the use of this interview tool enhanced person-centered care in clinical AR encounters. The audiologists highlighted that the tool facilitated patient participation and thus promoted a core concept of person-centered care. The use of the ICF-based holistic approach resulted in comprehensive patient narratives that guided the audiologists with their rehabilitation planning. Our findings revealed that the audiologists used concepts of person-centered care and that the ICF-based tool facilitated these efforts. To fully implement this tool into clinical practice, management staff may need to reorganize AR to foster a person-centered culture among clinicians and health care staff.

## Data Availability Statement

The raw data supporting the conclusions of this article will be made available by the authors, without undue reservation.

## Ethics Statement

Ethical review and approval was not required for the study on human participants in accordance with the local legislation and institutional requirements. Written informed consent for participation was not required for this study in accordance with the national legislation and the institutional requirements.

## Author Contributions

All authors listed have made a substantial, direct, and intellectual contribution to the work and approved it for publication.

## Funding

This project was funded by the Department of Audiology and the Faculty of Medicine and Health, Örebro University, Örebro.

## Conflict of Interest

The authors declare that the research was conducted in the absence of any commercial or financial relationships that could be construed as a potential conflict of interest.

## Publisher's Note

All claims expressed in this article are solely those of the authors and do not necessarily represent those of their affiliated organizations, or those of the publisher, the editors and the reviewers. Any product that may be evaluated in this article, or claim that may be made by its manufacturer, is not guaranteed or endorsed by the publisher.
